# Editorial: Mitochondrial Metabolism in Ischemic Heart Disease

**DOI:** 10.3389/fcvm.2022.961580

**Published:** 2022-06-27

**Authors:** Lei Yang, Shijun Wang, Jian Wu, Lei-Lei Ma, Yang Li, Haiyang Tang

**Affiliations:** ^1^College of Veterinary Medicine, Northwest A&F University, Xianyang, China; ^2^Shanghai Institute of Cardiovascular Diseases, Zhongshan Hospital and Institutes of Biomedical Sciences, Fudan University, Shanghai, China; ^3^Department of Cardiology, Zhongshan Hospital, Fudan University, Shanghai, China; ^4^Department of Ophthalmology, Stanford University, Palo Alto, CA, United States; ^5^State Key Laboratory of Respiratory Disease, National Clinical Research Center for Respiratory Disease, Guangzhou, China; ^6^Guangdong Key Laboratory of Vascular Disease, Guangzhou Institute of Respiratory Health, The First Affiliated Hospital of Guangzhou Medical University, Guangzhou, China

**Keywords:** mitochondria, heart failure, oxidative stress, cell metabolism, ischemia—reperfusion

Ischemic heart disease (IHD) is the single greatest cause of mortality worldwide and the most common underlying cause of heart failure (HF) (1). The major causes of IHD include atherosclerosis-related narrowing or obstruction of the vascular lumen, myocardial ischemia, and hypoxia/necrosis caused by coronary artery spasm. Despite existing treatments and interventions such as drug therapy, left ventricular assist device, artificial heart, and heart transplantation, the progression of HF caused by IHD is essentially irreversible (2, 3). Underlying the pathogenic mechanisms of IHD lacks comprehensively understanding and the investigations on the novel targets and signaling pathways involved in the development and progression of IHD are urgently needed.

Mitochondria are critical in the energy production of cardiomyocytes, generating 90% of adenosine triphosphate (ATP) for cardiac energy support. Moreover, mitochondria are the main sites for generating oxidative phosphorylation (OXPHOS), which is the primary energy source for maintaining normal heart contractile function (4). Mitochondria are not only the power plants of the cardiomyocytes, but also is the centers of signal transmission (5, 6), and they are closely connected with cell activities such as apoptosis (7), reactive oxygen species (ROS) generation (8), and lipid metabolism (9). Nevertheless, how aberrant regulation of mitochondria functions as an antecedent indicator of IHD and its restoration by pharmaceutical or non-pharmaceutical intervention is yet to elucidate.

Recent reports including the studies under this Research Topic have borne out claims that mitochondrial dysfunction is highly associated with IHD. Contributing factors to mitochondrial disorder in IHD include mitophagy disorder, ROS exacerbation and mitochondria-related inflammation. Xin et al. reported that mitochondria are important organelles involved in energy production of cardiomyocytes, mitochondrial disorder results in the pathogenesis of IHD.

Oxidative stress, caused by excessive ROS, is a crucial pathophysiological mechanism of myocardial infarction (MI), through impairment of cell function and vitality. ROS are byproducts of the mitochondrial synthesis of ATP. When mitochondrial function declines, more ROS are produced, resulting in the development of MI (10). Shen et al. found that dioscin, a natural product, alleviated cardiac dysfunction and remodeling post-MI by maintaining the Kreb's cycle and restraining mitochondrial ROS accumulation to improve mitochondrial quality control. Hence, restoring mitochondrial function may be an effective therapeutic strategy for IHD. Zhang et al. constructed novel biomimetic adipose-derived stem cells (ADSC)-derived nanovesicles (ADSC NVs) and consisted engineered ADSC NVs with melatonin (Mel) to form a novel Mel@NVs delivery system. The Mel@NVs alleviated mitochondrial dysfunction and promoted myocardial repair by ameliorating excessive ROS generation, promoting microvascular formation, and attenuating cardiac fibrosis. Therefore, targeting anti-ROS production would be a new therapeutic strategy for IHD.

Mitophagy is a kind of autophagy that removes damaged or dysfunctional mitochondria to prevent excessive accumulation of ROS. Mitochondria maintain the stability of mitochondrial dynamics through mitophagy, which is the basis of maintaining mitochondrial homeostasis and ensuring mitochondrial function. Proper functioning of this machinery is essential for maintaining a healthy cardiovascular system (11, 12). Uchikado et al. indicated oxidized low-density lipoprotein (ox-LDL) induces mitochondrial fission, leading to mitochondrial dysfunction and senescence through the association of lectin-like oxidized low-density lipoprotein scavenger receptor-1 (LOX-1) and angiotensin II type 1 receptor (AT1R), which activates the CRAF/MEK/ERK pathway. Furthermore, AT1R inhibition suppresses dynamin-related protein 1 (Drp1) activation and induces Rab9-dependent alternative autophagy through the CRAF/MEK/ERK axis in human vascular smooth muscle cells (VSMCs). Inhibition of AT1R also ameliorates cellular senescence by regulating mitochondrial dynamics, which may provide new ideas for the treatment of IHD.

As the second most energy-consuming organ of the body, the heart needs cardiomyocytes to produce a large amount of ATP to maintain normal functions, and most of the energy generated by cardiomyocytes comes from mitochondrial metabolism. In IHD, insufficient oxygen supply and mitochondrial dysfunction lead to reduced ATP production, making glycolysis as a more prominent source of energy (13). In this Research Topic collection, Jiang et al. summarized the role of abnormal mitochondrial metabolism in IHD progression and the current understanding of shutting down mechanotransduction to improve mitochondrial dysfunction and HF. Although increasing numbers of mechanical circulatory support (MCS) devices have been applied in clinical practice to alter myocardial energy metabolism, more research is needed to clarify its benefits on cardiac function and long-term prognosis. Guo et al. also found that cardiomyocyte-specific Neuraminidase1 (NEU1) deficiency alleviated MI-induced myocardial remodeling, oxidative stress, and mitochondrial metabolism disorder by regulating the sirtuin-1/peroxisome proliferator-activated receptor γ coactivator α (SIRT1/PGC-1α) signaling pathway. Cardiomyocyte-specific NEU1 inhibition provides a new direction for treatment after MI. Inhibitors of NEU1 may be a novel treatment option for IHD by ameliorating mitochondrial metabolism.

Moderate aerobic exercise is known to have a beneficial effect on cardiac function in HF (14). Cardiometabolic disorders in patients with HF can be partially recovered through exercise because of improved mitochondrial quality control, bioenergetics and cardiac function (15). Gu et al. in Research Topic, discussed the mechanism of aerobic exercise in maintaining normal mitochondrial function and improving cardiovascular disease. Mitofusin 2 (MFN2) and optic atrophy 1 (OPA1), which are beneficial to mitochondrial fusion and biogenesis, can be elevated by aerobic exercise. Drp1-induced mitochondrial fission can be inhibited by aerobic exercise in cardiovascular diseases. Regular aerobic exercise can reduce mitochondrial fission, enhance mitochondrial fusion, and improve mitochondrial autophagy or biogenesis, thus benefiting human health. Moreover, Yu et al. found exercise-generated β-Aminoisobutyric Acid (BAIBA) effectively improved both cardiac function and energy metabolism and reduced apoptosis in rats with HF after MI. BAIBA increased AMP-activated protein kinase (AMPK) phosphorylation through the expression of miR-208b and reduced apoptosis in H_2_O_2_-induced H_9_C_2_ cells, reshaping the energy metabolism of rats with HF and improving mitochondrial dysfunction. BAIBA has potential as an alternative to exercise therapy or synergistically with exercise therapy in the treatment of IHD.

In conclusion, this Research Topic discusses the role of mitochondria in the process of IHD and provides some strategies for mitochondria- targeted IHD therapy (Figure 1). Restoration of mitochondrial dynamics and metabolism is effective in alleviating experimental MI and HF, and further clinical trials are needed to confirm these strategies. Natural or artificial compounds that ameliorate excess ROS could reduce mitochondrial dysfunction and alleviate cardiac dysfunction, which may provide a novel treatment for IHD in the future. In addition, moderate aerobic exercise shows promise in restoring heart function in IHD, and maintaining regular exercise is also an effective strategy for preventing IHD.

**Figure 1:**
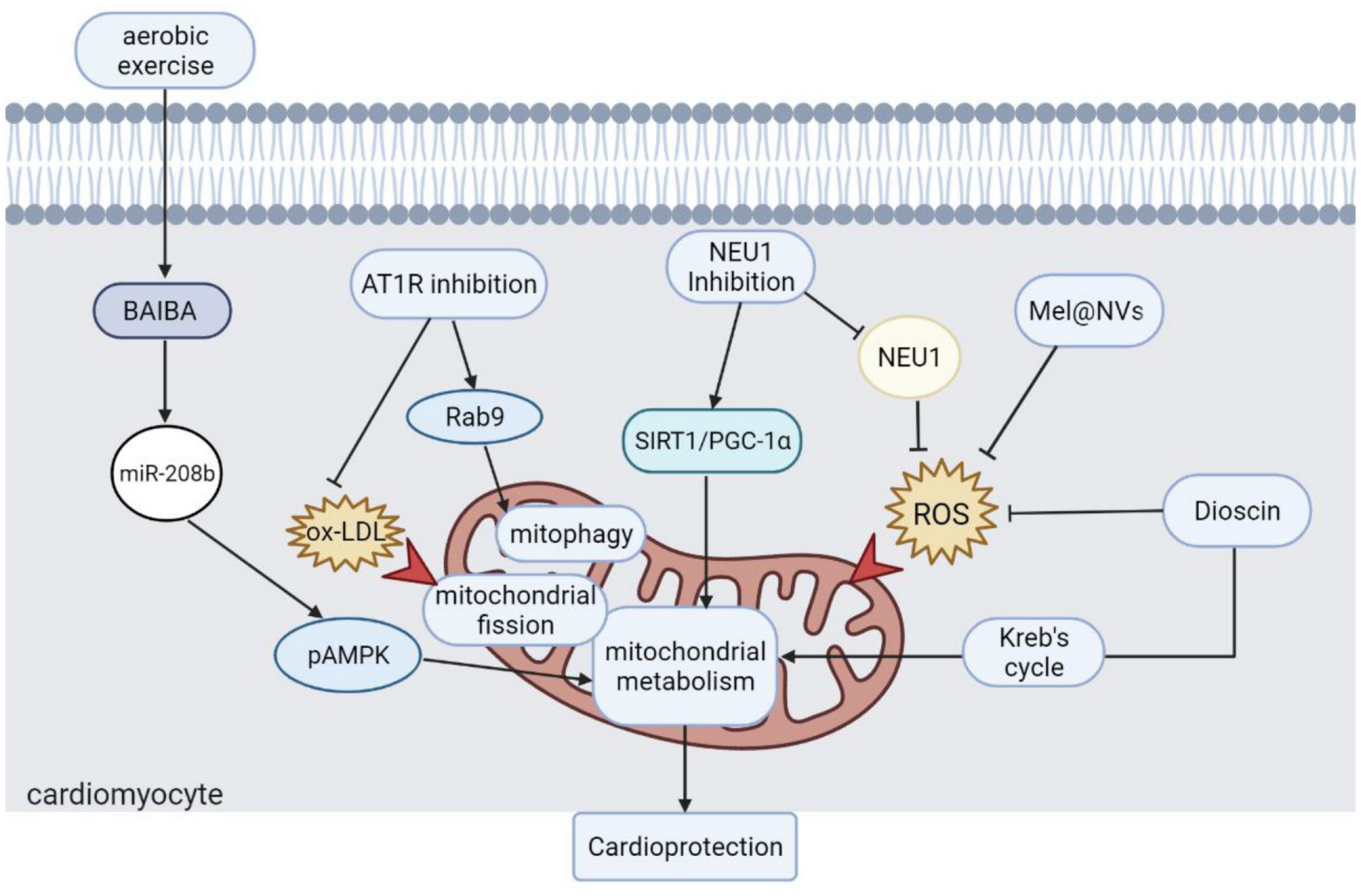
Strategies for mitochondria-targeted IHD therapy. Moderate aerobic exercise is an effective strategy to protect heart function. Different approaches to restore mitochondrial dynamics, autophagy, and metabolism are effective strategies for the treatment of IHD. Scavenging ROS accumulation is also one of the ways to restore mitochondrial function, which also plays a positive role in the recovery of cardiac function. BAIBA, β-Aminoisobutyric Acid; pAMPK, AMPK phosphorylation; AT1R, angiotensin II type 1 receptor; ox-LDL, oxidized low-density lipoprotein; Drp1, dynamin-related protein 1; NEU1, neuraminidase 1; SIRT1, sirtuin-1; PGC-1α, peroxisome proliferator-activated receptor γ coactivator α; Mel@NVs, melatonin engineered NVs; ROS: reactive oxygen species.

## Author Contributions

LY draft the manuscript with input from SW, JW, L-LM, YL, and HT. All authors contributed to the article and approved the submitted version.

## Funding

This work was supported in parts by National Key Research and Development Program of China (2019YFE0119400) and Natural Science Foundation of China (81970052 and 82170057).

## Conflict of Interest

The authors declare that the research was conducted in the absence of any commercial or financial relationships that could be construed as a potential conflict of interest.

## Publisher's Note

All claims expressed in this article are solely those of the authors and do not necessarily represent those of their affiliated organizations, or those of the publisher, the editors and the reviewers. Any product that may be evaluated in this article, or claim that may be made by its manufacturer, is not guaranteed or endorsed by the publisher.
